# Association between Periodontal Disease and Bone Loss among Ambulatory Postmenopausal Women: A Cross-Sectional Study

**DOI:** 10.3390/jcm13195812

**Published:** 2024-09-28

**Authors:** Sophie Ahmad, Nataliyah Tahir, Rafae Nauman, Ashok Gupta, Civon Gewelber, Kavita Batra, Kenneth Izuora

**Affiliations:** 1 Kirk Kerkorian School of Medicine at UNLV, University of Nevada Las Vegas, 625 Shadow Lane, Las Vegas, NV 89106, USA; ahmads1@unlv.nevada.edu (S.A.); tahirn1@unlv.nevada.edu (N.T.); naumar1@unlv.nevada.edu (R.N.); 2 Greensboro Radiology, 1331 N Elm Street, Greensboro, NC 274402, USA; ashok.gupta@radpartners.com; 3 College of Dental Medicine, Roseman University of Health Sciences, 1 Breakthrough Way, Suite 3218, Las Vegas, NV 89135, USA; cgewelber@roseman.edu; 4 Department of Medical Education and Office of Research, Kirk Kerkorian School of Medicine at UNLV, University of Nevada Las Vegas, 1701 W Charleston Blvd, Suite 200-07, Las Vegas, NV 89102, USA; kavita.batra@unlv.edu; 5 Department of Internal Medicine, Kirk Kerkorian School of Medicine at UNLV, University of Nevada Las Vegas, 1701 W Charleston Blvd, Suite 230, Las Vegas, NV 89102, USA

**Keywords:** osteoporosis, osteopenia, periodontal disease, inflammation

## Abstract

**Background/Objectives**: Osteoporosis and periodontal disease (PD) are associated with significant morbidity and mortality especially among post-menopausal women. The attributable causes of mortality include bone fragility, hip fractures, surgical risks, complications associated with immobility/disability, and mental health issues. This cross-sectional study aims to investigate the association between oral health and bone diseases along with the factors that predict this association. **Methods**: This study included post-menopausal women undergoing routine bone density evaluation. Following informed consent, case histories were collected using an investigator-administered questionnaire. The oral cavity was inspected for the health of the oral structures and periodontium. Bone density data, interpreted by a radiologist, were also collected. Data were analyzed using chi-square and logistic regression tests with the significance level set at 5%. **Results**: Among 100 eligible participants, mean age and body mass index (BMI) were 68.17 ± 8.33 years and 29.59 ± 6.13 kg/m^2^, respectively. A total of 23 participants (23.0%) had T2DM, 29 (29.0%) had < 20 natural teeth, and 17 (17.0%) had normal bone mineral density. Except for age (aOR 1.171, *p* < 0.001), BMI (aOR 0.763, *p* < 0.001), and past osteoporotic fractures (aOR 21.273, *p* = 0.021), all other factors were insignificant predictors of bone loss. **Conclusions**: Although the unadjusted results suggest a relationship between oral health indicators and bone loss, these relationships were not present when other factors were included in an adjusted model. Our findings suggest PD by itself may not be a risk factor for bone loss but that the two conditions may have similar risk factors.

## 1. Introduction

Menopause represents a critical physiological transition in women, marked by the cessation of menstruation and significant hormonal changes. This transition is associated with a range of health challenges, among which significant bone loss leading to osteoporosis (OP) and worsening oral health, particularly periodontal disease (PD), are of paramount concern [1,2]. The implications of these health issues extend beyond physical changes and discomfort, as they contribute to significant morbidity and mortality among postmenopausal women. The attributable causes of mortality include, but may not be limited to, bone fragility, hip fractures, surgical risks, complications associated with immobility/disability, and mental health issues [3,4,5].

OP involves loss of bone mineral density that results in decreased bone strength, predisposing affected individuals to fractures. OP is often referred to as a “silent disease” because it progresses without symptoms until a fragility fracture occurs as its first clinical presentation, making it imperative to understand and address the factors that contribute to its development [6]. Better understanding of the factors that predict or are associated with OP-related fractures will contribute to efforts to reduce the burden of this condition.

PD, a common condition characterized by gum inflammation and degeneration of supporting structures of the teeth, is another significant concern in postmenopausal women. PD is caused by the accumulation of plaque and tartar on the teeth, leading to inflammation of the gums which ultimately causes alveolar bone loss, tooth mobility, and subsequent tooth loss. The consequences of PD are not limited to the oral cavity; it has been linked to several systemic conditions, such as uncontrolled diabetes mellitus, cardiovascular diseases, and respiratory diseases [7,8,9,10]. These adverse effects are hypothesized to be related to PD causing an increase in systemic inflammation [8,11,12]. Recent studies have also suggested a potential link between PD and bone loss, leading to an increased fracture risk [13,14]. This is particularly concerning in postmenopausal women, who are already at increased risk for osteoporosis.

Recent systematic reviews and meta-analyses have also found that postmenopausal women with OP are more likely to have periodontitis, suggesting routine evaluation of OP in those found to have PD [13,14]. The proposed mechanism linking PD and OP involves systemic inflammation, which is thought to exacerbate both conditions. Chronic inflammation, a hallmark of periodontitis, is known to increase the production of pro-inflammatory mediators, which can lead to a dysregulated immune-mediated inflammatory response, ultimately damaging teeth and their supporting structures [7,11,12]. A dysregulated inflammatory response is also hypothesized to be a contributing factor to bone loss and OP [14]. Understanding how these shared risk factors contribute to the co-occurrence of PD and OP is essential for developing integrated prevention and treatment strategies.

The implications of the connection between OP and PD are considerable, given that the economic burden of both OP and PD is staggering, not only due to medical costs but also because of the long-term care required for individuals who suffer from fractures and complications of PD [3,4,5]. Hip fractures, in particular, are associated with high mortality rates, prolonged disability, and significant healthcare costs [3,4]. These fractures often lead to a decline in quality of life, with many patients experiencing a loss of independence. Additionally, patients with PD are burdened with oral complications and treatment costs [5]. The indirect costs of these diseases, including loss of productivity and the need for caregiving, further exacerbate the economic impact on both families and the healthcare system as a whole [3,4,5].

Despite the prevalent evidence suggesting an association between OP and PD, there is inconclusive evidence to explain the relationship between these two conditions. Moreover, the underlying mechanism for a potential causal relationship between these two conditions remains unclear. It is known that OP and PD share several common risk factors, such as advancing age, diabetes mellitus, smoking and alcohol consumption, and calcium deficiency [15]. Understanding the associations between OP and PD in the context of these risk factors will help better understand their pathophysiology. This could potentially facilitate their earlier detection and efforts to reduce their prevalence and improve outcomes in this population.

This study seeks to address a specific gap in the literature by exploring the relationship between PD and OP in a population of ambulatory postmenopausal women. This population is particularly vulnerable to both conditions, yet it has been relatively understudied in this context. By examining the intersection of these two health issues, the study aims to identify factors that contribute to their co-occurrence and to explore potential protective factors that could mitigate their impact.

Against this backdrop, given the significant public health implications of both conditions, a clearer understanding of their association could lead to new insights into their pathophysiology and to the development of more effective strategies for their prevention and management. By focusing on a population that is at high risk for both PD and OP, this study aims to contribute to a more holistic understanding of how these conditions interact and to inform the development of integrated care approaches that improve the overall health and well-being of postmenopausal women.

## 2. Materials and Methods

### 2.1. Study Design and Study Setting

This was a cross-sectional, single institutional study conducted at an ambulatory diagnostic radiology center located in an urban setting. Consecutive individuals presenting for routine bone density evaluation were approached for enrollment. Following an explanation of the study procedure, informed consent was obtained from eligible individuals who agreed to participate in the study. This study was approved and overseen by the UNLV Institutional Review Board (IRB number 1597219-1) and was conducted with appropriate ethical standards for the protection of study participants.

### 2.2. Inclusion/Exclusion Criteria

Subjects included were postmenopausal women who were 50 years or older, referred for screening for osteoporosis and osteopenia, and able to understand and give informed consent. We excluded those with advanced debilitating conditions and those who were unable to provide consent. We used an age cutoff of 50 years, as many major epidemiological studies looking at interventions in post-menopausal women, such as the Women’s Health Initiative, have used similar inclusion criteria to define post-menopausal status [16].

### 2.3. Study Measures

**Figure 1:**
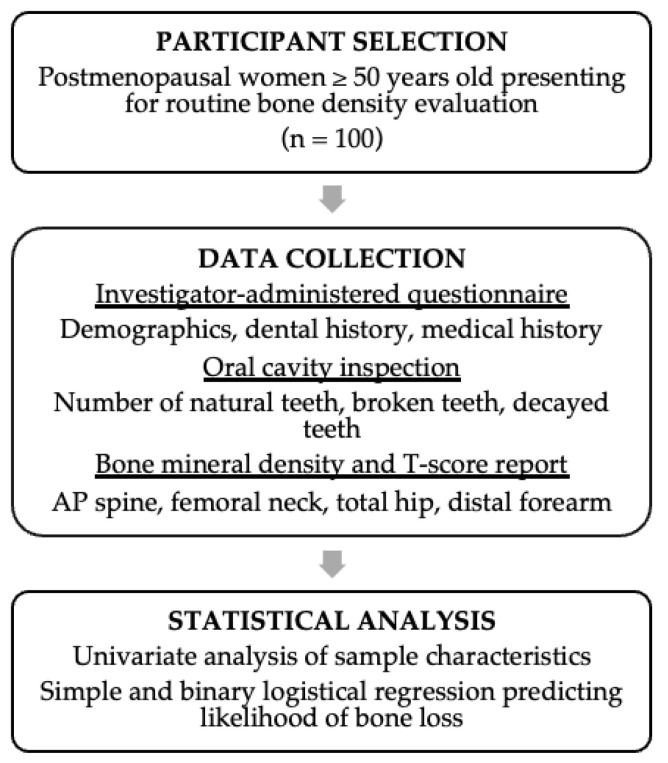
Summary of study design and methodology.

Following enrollment and informed consent, we gathered information on participants’ demographics, dental history, and medical history using an investigator-administered questionnaire (Appendix A). Although this questionnaire is not standardized, questions were adapted from standardized tools and previous research showing the validity of questionnaires to screen for PD [17]. Additional questions were formulated based on known predictors of OP and PD. This approach is very important in epidemiological research involving PD, given that not every patient has access to a formal dental evaluation. Height and weight were measured, and we inspected their oral cavities to count the number of teeth and to document the number of broken or decayed teeth present. Prior to beginning data collection, the study team was trained and calibrated on proper dental examination techniques by a dental expert (CG). This involved reviewing images and criteria required to define teeth as “normal”, “decayed”, “broken”, and “missing” based on physical inspection. Teeth were categorized as “broken” if any portion, but not the entire tooth, was missing. Tooth “decay” was defined as having discoloration or breakdown of tooth enamel on inspection. Teeth were categorized as “missing” if all parts of the tooth, including the root, were absent. Finally, teeth were categorized as “normal” if the above criteria were absent and the teeth appeared to be intact. All study assessments, including the bone density evaluation, periodontal evaluation, and questionnaire, were collected on the same day for each participant. Participant bone mineral density reports were obtained after interpretation by the radiologist, and bone mineral density and T-scores at the AP spine, femoral neck, total hip, or distal forearm were collected. Participants were classified either as having normal bone mineral density (BMD) or bone loss based on BMD in the osteopenia or osteoporotic range [18]. PD was defined as an affirmative response to any one of the following: history of gum disease, receipt of deep cleaning, loose teeth, tooth sensitivity, or gum bleeding on the questionnaire [19,20,21]. The number of remaining teeth were categorized into ≥20 and <20 teeth as described by a previous study [22]. BMI was categorized using the standards provided by the Centers for Disease Control and Prevention (CDC) [23]. Figure 1 describes the eligibility criteria, data collection, and statistical approach.

### 2.4. Sample Size Justification

For the power analysis, we used G power software (version 3.1) with large Cohen’s effect size conventions [24,25,26]. For the logistic regression analysis, we used the formula proposed by Green (N ≥ 50 + 8 m, where m corresponds to the number of predictors) [26]. The total number of predictors was 9, according to which, N = 122 was deemed appropriate. The total sample size estimated with a power of 0.80 was 88 (effect size = 0.3) for the chi-square test. The sample size with the greatest value (N = 122) was considered appropriate since it satisfies the minimum requirement of all the statistical tests used. The sample used in this study was underpowered, which we acknowledged in the limitation section.

### 2.5. Statistical Analysis

First, a univariate analysis was performed to understand the distribution of the data. Categorical variables were represented as frequencies and proportions, whereas continuous variables were represented by the mean and standard deviations. The chi-square/Fisher exact test was used to compare the nominal groups. For the regression analysis, an initial adjusted model was used to generate crude estimates. Estimates for the parameters were obtained through the maximum likelihood estimation method with 95% Wald’s confidence limits for the logistic model. The final model was selected based upon the Akaike information criterion (AIC) and the Schwarz Criterion (SC) [27]. A binary logistic regression (final model) was performed to model the probability of bone loss after adjusting variables, such as age, BMI, race/ethnicity, T2DM, number of decayed and broken teeth, presence of periodontal diseases and osteoporotic fractures through calculating adjusted odds ratios. The 95% CIs for effect sizes in the chi-square tests were calculated manually by using the calculated standard error, margin of error, and sample size. The significance level was set at 0.05. All analyses were conducted using SPSS version 27 and SAS 9.4 [28,29].

## 3. Results

In a sample of 100 eligible post-menopausal women, the mean age and body mass index (BMI) were 68.17 ± 8.33 years and 29.59 ± 6.13 kg/m^2^, respectively. Twenty-three participants (23.0%) had T2DM, 29 (29.0%) had less than 20 natural teeth, and 17 (17.0%) had normal bone mineral density (Table 1).

Among the subjects with normal bone density, a significantly higher proportion of subjects were obese (65.3% vs. 32.5%, *p* = 0.04, effect size = 0.3 [95% CI: 0.23–0.34]), had type 2 diabetes mellitus (39.1% vs. 18.2%, *p* = 0.036, effect size = 0.2 [95% CI: 0.16–0.24]), and were current smokers (13.0% vs. 2.6%, *p* = 0.044, effect size = 0.2 [95% CI: 0.16–0.24]), as opposed to those with bone loss (Table 2). On the contrary, subjects with bone loss had a higher proportion of osteoporotic fractures as opposed to their counterparts with normal bone density (28.6% vs. 4.3%, *p* = 0.015, effect size = 0.2 [95% CI: 0.16–0.24]); Table 2).

In an unadjusted simple logistic regression, age, BMI, race/ethnicity, number of natural teeth, history of PD, and history of osteoporotic fracture were associated with bone loss. However, in the binary logistic regression, the only significant predictors of bone loss included age (aOR 1.171 [95% CI:1.082–1.268], *p* < 0.001), BMI (aOR 0.763 [95% CI: 0.656–0.888], *p* < 0.001), and history of osteoporotic fractures (aOR 21.273 [95% CI: 1.601–282.71] *p* = 0.021; Table 3).

## 4. Discussion

In the unadjusted analysis, age, BMI, race/ethnicity, number of natural teeth, PD, and past osteoporotic fracture were associated with bone loss. However, in the adjusted analysis, the only significant predictors of bone loss were age, BMI, and past osteoporotic fractures.

Osteoporosis and PD are two conditions that result in adverse health outcomes. Understanding the relationship between PD and bone loss associated with OP is important since this could help predict individuals at risk for both conditions and potentially allow for earlier detection, intervention, and treatment. This is especially important among postmenopausal women who are at increased risk for both conditions [30,31]. To better understand the relationship between these two conditions, our study looked at the distribution of common risk factors among post-menopausal women and found that the expected risk factors (older age, low BMI, and prior OP fracture) predicted bone loss and that although PD was associated with bone loss in the unadjusted regression model, when accounting for other risk factors in the regression model, PD did not predict bone loss.

The relationship between age and bone health has been widely studied, with a consensus that increasing age is associated with increasing bone loss. After the achievement of peak bone mass around the age of 30 years, bone remodeling results in a net bone loss with advancing age and with the onset of menopause in women [32,33,34]. Our study found similar results, with age being a significant risk factor of bone loss in a post-menopausal population.

Additionally, we found that lower BMI is a significant predictor of bone loss. This is consistent with previously published studies. One cross-sectional study found that BMI was inversely associated with bone loss and that women with a low BMI are at increased risk of osteoporosis [35]. Laet et al. in a meta-analysis concluded that a low BMI conferred an increased fracture risk independent of age and gender [36,37]. Another study among a cohort of 1902 racially diverse women found significant reduction of bone density, especially during late menopause, and that body weight was a major determinant of the rate of menopause-related bone loss, independent of ethnicity [34,38]. We found similar results in our study, with a significant association between bone loss and BMI, but not with ethnicity. Also, with the average age of menopause being around 51 years, our sample, with an average age of 68 years of age, likely represents women in the late post-menopausal stage, which could explain the similarity between our findings and previously published studies [39].

The association between bone loss with OP fractures has been well described by previous studies. This association is likely due to the underlying pathophysiology of osteoporosis where reduced bone mineral density results in weakened bone strength, predisposing to OP-related fragility fractures with minimal trauma [40,41]. Our observation of this association is therefore anticipated and is consistent with previous research.

While some significant predictors of bone loss aligned with those found in the previously published literature, in our study, the binary logistic regression model revealed that oral health measures including PD were not significant predictors of bone loss. Previous research attempting to understand this relationship has shown some association between the two conditions, which has been theorized to be due to bone loss in the jaw which predisposes to PD and tooth loss [42,43]. Another study showed a clear association between low BMD and periodontitis, but only in women above 58 years of age and independent of tobacco consumption or oral hygiene [44]. An analysis of the National Health and Nutrition Examination Survey (NHANES) study also found an association between OP and periodontitis especially in post-menopausal women [31]. On the other hand, other research suggests that the two conditions (bone loss and PD) do not have a causal relationship and that the association between them may be a result of similarities in their risk factors [45]. Such risk factors include age, inadequate dietary calcium intake, smoking, and post-menopausal status. Also, the presence of systemic inflammation may be a common finding for these two conditions and another shared risk factor. Our findings are more consistent with the latter theory, where the initial association we found was not apparent in a bivariate logistic regression model accounting for the other variables, including the shared risk factors.

### Strengths and Limitations

While the relationship between osteoporosis and periodontal disease has been previously studied, the focus on ambulatory post-menopausal women adds an original element. This demographic is often underrepresented in research, making this study valuable for filling a gap in existing knowledge [46,47,48]. By focusing on a specific and vulnerable population, the study could lead to the development of targeted preventive strategies or interventions that address both osteoporosis and periodontal disease concurrently, which is a relatively unexplored area [46,47,48]

However, this study is not without limitations. First, given the single-institutional nature of this study, the external validity will be limited, which will not allow generalization of these findings to other settings and populations. Second, due to the cross-sectional nature of this study, cause–effect relationships could not be determined, so the temporal sequence between exposure and outcome could not be investigated. Additionally, we acknowledge that our study population may have other undiagnosed bone conditions and limited awareness of these conditions. These potential confounding conditions were unable to be identified or controlled at the time of enrollment. Next, there can be a possibility of measurement bias, especially in the case of quantifying the health of the periodontium and oral structures. Future studies may consider using the standardized periodontal indices (i.e., Russell’s periodontal index, Gingival bone count index, and community periodontal index for treatment needs), dental health indicators (i.e., Decayed, Missing, Filled Teeth [DMFT] index), and several others [49,50]. Additionally, this study may be prone to a residual confounding bias due to some factors, such as bruxism being unmeasured [51,52]. Lastly, this study was underpowered, with a minimum sample requirement of 122 vs. the given sample of 100 subjects. However, effect sizes obtained from this study will be used to estimate the sample size for a larger prospective study that will also account for several other limitations noted in this study.

## 5. Conclusions and Clinical Implications

Clinical variables associated with bone loss in our study included older age, lower BMI, and history of osteoporotic fracture. These are established risk factors for bone loss. Although unadjusted results suggest a relationship between oral health measures and bone loss, these relationships were not present when other factors were included in a multivariate model. These findings suggest that PD by itself may not have a direct causal relationship with bone loss but rather, may reflect the similarity in the risk factors for both conditions. Future well-designed prospective studies are needed to explore a potential causal relationship between bone loss and PD.

This study has the potential to significantly influence clinical practice by promoting more integrated, preventive (coordinated screenings), and personalized care approaches (i.e., developing risk assessment tools that consider OP and PD) for post-menopausal women. It could lead to new guidelines, improved patient outcomes, and a more holistic understanding of the interconnections between systemic and oral health. For example, dentists might become more vigilant in screening for osteoporosis in patients presenting with periodontal disease, and vice versa, leading to earlier detection and intervention for both conditions. Understanding the relationship between these conditions might result in new preventive strategies, such as recommending bone density tests for patients with severe periodontal disease or suggesting more rigorous oral hygiene practices for patients with osteoporosis.

## Figures and Tables

**Table 1:** Descriptive statistics of study population (N = 100).

Variable Name	Categories	Statistics
		
Age in years (M ± SD)	-	68.17 ± 8.330
BMI (M ± SD)	-	29.59 ± 6.13
BMI categories	Underweight	3 (3.0)
	Normal	19 (19.0)
	Overweight	38 (38.0)
	Obese	40 (40.0)
Race/Ethnicity	White	47 (47.0)
	Black or African American	19 (19.0)
	Hispanic or Latino	23 (23.0)
	Other	11 (11.0)
T2DM	Yes	23 (23.0)
	No	77 (77.0)
Natural teeth group	Group 1 (≥20)	71 (71.0)
	Group 2 (<20)	29 (29.0)
Broken teeth (n = 58)	Yes	23 (23.0)
	No	77 (77.0)
Decayed teeth (n = 58)	Yes	29 (29.0)
	No	71 (71.0)
How many times a week do you brush? (M ± SD)	-	12.845 ± 4.685 0, 21.0
How many times a week do you floss? (M ± SD)	-	5.82 ± 4.980 0, 21.0
How many times a week do you use mouthwash (M ± SD)	-	5.16 ± 6.051 0, 21.0
Periodontal disease	Yes	81 (81.0)
	No	19 (19.0)
Rate the health of your teeth	Excellent	12 (12.0)
	Very good	23 (23.0)
	Good	35 (35.0)
	Fair	21 (21.0)
	Poor	9 (9.0)
OP fracture	Yes	23 (23.0)
	No	77 (77.0)
Are you taking steroid medication?	Yes	11 (11.0)
	No	89 (89.0)
Rheumatoid arthritis	Yes	10 (10.0)
	No	90 (93.9)
Current smoker	Yes	5 (5.0)
	No	95 (95.0)
Past smoker	Yes	39 (39.0)
	No	57 (57.0)
Bone	Normal	17 (17.0)
	Osteopenia	51 (51.0)
	Osteoporosis	26 (26.0)

Note: Statistics are reported as frequency and proportion unless stated otherwise. M = Mean; SD = Standard deviation.

**Table 2:** Comparing characteristics of the sample (N = 100).

Variable Name	Categories	Normal n = 23 (23.0)	Osteopenia/Osteoporosis n = 77 (77.0)	*p* Value	Chi-Square Statistic	Effect Size
BMI categories	Underweight	0 (0.0)	3 (3.9)	**0.04**	8.281	0.3
	Normal	3 (13.0)	16 (20.8)			
	Overweight	5 (21.7)	33 (42.9)			
	Obese	15 (65.3)	25 (32.5)			
Ethnicity	White	8 (34.8)	39 (50.6)	0.15	5.387	0.2
	Black or African American	8 (34.8)	11 (14.3)			
	Hispanic or Latino	4 (17.4)	19 (24.7)			
	Other	3 (13.0)	8- (10.4)			
T2DM	Yes	9 (39.1)	14 (18.2)	**0.036**	4.388	0.2
	No	14 (60.9)	63 (81.8)			
Natural teeth	Group 1 (≥20)	16 (69.6)	55 (71.4)	0.863	0.030	0.02
	Group 2 (<20)	7 (30.4)	22 (28.6)			
Broken teeth	Yes	8 (34.8)	15 (19.5)	0.126	2.342	0.5
	No	15 (65.2)	62 (80.5)			
Decayed teeth	Yes	11 (47.8)	18 (23.4)	**0.023**	5.142	0.2
	No	12 (52.2)	59 (76.6)			
Periodontal disease	Yes	20 (87.0)	61 (79.2)	0.407	0.689	0.08
	No	3 (13.0)	16 (20.8)			
Rate your teeth	Excellent	2 (8.7)	10 (13.0)	0.956	0.664	0.08
	Very good	5 (21.7)	18 (23.3)			
	Good	8 (34.8)	27 (35.1)			
	Fair	6 (26.1)	15 (19.5)			
	Poor	2 (8.7)	7 (9.1)			
OP fracture	Yes	1 (4.3)	22 (28.6)	**0.015**	5.868	0.2
	No	22 (95.7)	55 (71.4)			
Are you taking steroid	Yes	1 (4.3)	10 (13.0)	0.245	1.350	0.1
	No	22 (95.7)	67 (87.0)			
Rheumatoid arthritis	Yes	1 (4.3)	9 (11.7)	0.303	1.060	0.1
	No	22 (95.7)	68 (88.3)			
Current smoker	Yes	3 (13.0)	2 (2.6)	**0.044**	4.068	0.2
	No	20 (87.0)	75 (97.4)			

*p* values less than 0.05 (in bold text) are considered statistically significant.

**Table 3:** Logistic regression predicting likelihood of bone loss based on selected independent variables (N = 100).

Variable	Unadjusted Results				Adjusted Results			
Categories	Odds Ratio	*p* Value	LCL	UCL	Odds Ratio	*p* Value	LCL	UCL
Age	1.109	**<0.001**	1.012	1.026	1.171	**<0.001**	1.082	1.268
BMI	1.034	**<0.001**	1.019	1.050	0.763	**<0.001**	0.656	0.888
Race/Ethnicity—White (ref: Black)	4.875	**<0.001**	2.278	10.432	2.181	0.394	0.363	13.121
Hispanic or Latino	4.750	**0.005**	1.616	13.962	7.048	0.055	0.960	51.740
Others	2.667	0.147	0.707	10.052	1.073	0.953	0.104	11.10
T2DM (Re: No)	1.556	0.301	0.673	3.594	0.360	0.221	0.070	1.848
Natural teeth (ref: Group 1 ≥20)	3.143	**0.008**	1.343	7.357	0.388	0.214	0.087	1.727
Broken teeth (ref: N)	1.875	0.151	0.795	4.422	0.264	0.100	0.054	1.291
Decayed teeth (ref: N)	1.636	0.198	0.773	3.465	0.597	0.454	0.155	2.302
Periodontal disease (ref: N)	3.050	**<0.001**	1.841	5.054	0.292	0.225	0.040	2.131
OP fracture (ref: N)	22.00	**0.003**	2.965	163.21	21.273	**0.021**	1.601	282.71

*p* values less than 0.05 (in bold text) are considered statistically significant.

## Data Availability

The original contributions presented in the study are included in the article, further inquiries can be directed to the corresponding authors.

## Appendix A. Questionnaire

RELATIONSHIP BETWEEN ORAL HEALTH AND OSTEOPOROSIS AMONG AMBULATORY PATIENTS.		
**PI:** KENNETH IZUORA, MD **STUDY IDENTIFICATION NUMBER**: ____________ **DATE:** _____________		
DEMOGRAPHICS		
**GENDER** ☐ MALE ☐ FEMALE **AGE**:		
**HEIGHT**: ___________ ☐ FT ☐ CM **WEIGHT**: ___________ ☐ LBS ☐ KGS		
**ETHNICITY**	1. ☐ CAUCASIAN 2. ☐ HISPANIC OR LATINO 3. ☐ BLACK OR AFRICAN AMERICAN 4. ☐ ASIAN 5. ☐ AMERICAN INDIAN 6. ☐ FILIPINO 7. ☐ OTHER: _____________________________	
**LEVEL OF EDUCATION**	1. ☐ < HIGH SCHOOL GRADUATE 2. ☐ HIGH SCHOOL GRADUATE/SOME COLLEGE 3. ☐ COLLEGE DEGREE 4. ☐ MASTERS OR HIGHER DEGREE	
**TOTAL FAMILY INCOME**	A. ☐ < $12,490 B. ☐ $12,491–$16,910 C. ☐ $16,911–$21,330 D. ☐ $21,331–$25,750 E. ☐ $25,751–$30,170 F. ☐ $30,171–$34,590 G. ☐ $34,591–$39,010 H. ☐ $39,011–$43,430 I. ☐ $43,431–$47,850 J. ☐ $47,851–$52,270	K. ☐ $52,271–$56,690 L. ☐ $56,691–$60,000 M. ☐ $60,001–$70,000 N. ☐ $70,001–$80,000 O. ☐ $80,001–$90,000 P. ☐ $90,001–$100,000 Q. ☐ $100,001–$150,000 R. ☐ $150,001–$200,000 S. ☐ > $200,000
**NUMBER OF PEOPLE IN THE HOUSEHOLD**		

**DIABETES BACKGROUND**	
1. DO YOU HAVE DIABETES?	☐ YES ☐ NO
2. HOW LONG HAVE YOU HAD DIABETES?	___________ ☐ YEARS ☐ MONTHS
3. TYPE OF DIABETES	☐ TYPE 1 ☐ TYPE 2
2. ARE YOU TAKING INSULIN FOR YOUR DIABETES?	☐ YES ☐ NO

**CARDIOVASCULAR DISEASE**	
1. DO YOU HAVE ANY DAMAGE TO YOUR EYES FROM DIABETES?	☐ YES ☐ NO ☐ VERIFIED
2. DO YOU HAVE ANY DAMAGE TO YOUR KIDNEYS FROM DIABETES?	☐ YES ☐ NO ☐ VERIFIED
3. DO YOU HAVE ANY DAMAGE TO YOUR NERVES FROM DIABETES?	☐ YES ☐ NO ☐ VERIFIED
4. DO YOU HAVE HISTORY OF HEART DISEASE?	☐ YES ☐ NO ☐ VERIFIED
5. DO YOU HAVE HISTORY OF STROKE?	☐ YES ☐ NO ☐ VERIFIED

**DENTAL CARE**			
1. HOW MANY OF YOUR NATURAL TEETH DO YOU STILL HAVE IN YOUR MOUTH?	**TOTAL**	**BROKEN**	**DECAYED**
		
2. IN THE PAST 2 YEARS, HOW MANY TIMES HAVE YOU SEEN A DENTIST?			
3. HOW MANY TIMES A WEEK DO YOU BRUSH?			
4. HOW MANY TIMES A WEEK DO YOU FLOSS?			
5. HOW MANY TIMES A WEEK DO YOU USE MOUTHWASH?			
6. HAVE YOU EVER BEEN TOLD YOU HAVE GUM DISEASE?	☐ YES ☐ NO		
7. HAVE YOU EVER HAD TREATMENT FOR GUM DISEASE OR “DEEP CLEANING”?	☐ YES ☐ NO		
8. HAVE YOU BEEN TOLD BY A DENTAL PROVIDER THAT YOU LOST BONE AROUND YOUR TEETH?	☐ YES ☐ NO		
9. IN PAST 3 MONTHS, HAVE YOU NOTICED ANY TOOTH THAT DOESN’T LOOK RIGHT?	☐ YES ☐ NO		
10. DO YOU HAVE ANY LOOSE TEETH?	☐ YES ☐ NO		
11. DO YOU HAVE ANY TOOTH SENSITIVITY?	☐ YES ☐ NO		
12. DOES YOUR GUM BLEED SOMETIMES WHEN YOU BRUSH?	☐ YES ☐ NO		
OVERALL, HOW DO YOU RATE THE HEALTH OF YOUR TEETH? *CIRCLE ONE* EXCELLENT VERY GOOD GOOD FAIR POOR			

**BONE DISEASE**	
1. HAVE YOU BROKEN ANY BONE(S) IN THE PAST?	☐ YES ☐ NO
2. HOW OLD WERE YOU WHEN YOU BROKE YOUR BONE?	__________ YEARS OLD
3. HOW DID YOU BREAK YOUR BONE?	
4. DO YOU TAKE ANY OF THE FOLLOWING?	☐ CALCIUM SUPPLEMENT ☐ VITAMIN D ☐ MILK ☐ YOGURT ☐ CHEESE

**OTHER INCLUSION/EXCLUSION INFORMATION**	
1. ARE YOU TAKING STEROID MEDICATION?	☐ YES ☐ NO
2. ON AVERAGE, HOW MANY DRINKS OF ALCOHOL DO YOU HAVE PER DAY?	____________
3. HAVE YOU BEEN DIAGNOSED WITH RHEUMATOID ARTHRITIS?	☐ YES ☐ NO
4. DO YOU SMOKE CIGARETTES?	☐ YES ☐ NO
5. HAVE YOU SMOKED CIGARETTES IN THE PAST?	☐ YES ☐ NO
6. IF YES, WHEN DID YOU QUIT?	_______________________
7. IF FEMALE, ARE YOU POST MENOPAUSAL?	☐ YES ☐ NO
